# Neuroprotective mechanisms of luteolin in glutamate-induced oxidative stress and autophagy-mediated neuronal cell death

**DOI:** 10.1038/s41598-024-57824-2

**Published:** 2024-04-02

**Authors:** Wudtipong Vongthip, Sunita Nilkhet, Kanokkan Boonruang, Monruedee Sukprasansap, Tewin Tencomnao, Seung Joon Baek

**Affiliations:** 1 Department of Clinical Chemistry, Faculty of Allied Health Sciences, Program in Clinical Biochemistry and Molecular Medicine, Chulalongkorn University, 10330 Bangkok, Thailand; 2 Laboratory of Signal Transduction, Research Institute for Veterinary Science, College of Veterinary Medicine, Seoul National University, 1 Gwanak-ro, Gwanak-gu, Seoul, 08826 Korea; 3 Food Toxicology Unit, Institute of Nutrition, Mahidol University, Nakhon Pathom, 73170 Thailand; 4 Natural Products for Neuroprotection and Anti-Ageing Research Unit, Chulalongkorn University, Bangkok, 10330 Thailand

**Keywords:** Neuroprotection, Luteolin, Autophagy, Mitophagy, mTORC1 signaling pathway, Chemical biology, Neuroscience, Biomarkers, Molecular medicine

## Abstract

Neurodegenerative diseases, characterized by progressive neuronal dysfunction and loss, pose significant health challenges. Glutamate accumulation contributes to neuronal cell death in diseases such as Alzheimer's disease. This study investigates the neuroprotective potential of *Albizia lebbeck* leaf extract and its major constituent, luteolin, against glutamate-induced hippocampal neuronal cell death. Glutamate-treated HT-22 cells exhibited reduced viability, altered morphology, increased ROS, and apoptosis, which were attenuated by pre-treatment with *A. lebbeck* extract and luteolin. Luteolin also restored mitochondrial function, decreased mitochondrial superoxide, and preserved mitochondrial morphology. Notably, we first found that luteolin inhibited the excessive process of mitophagy via the inactivation of BNIP3L/NIX and inhibited lysosomal activity. Our study suggests that glutamate-induced autophagy-mediated cell death is attenuated by luteolin via activation of mTORC1. These findings highlight the potential of *A. lebbeck* as a neuroprotective agent, with luteolin inhibiting glutamate-induced neurotoxicity by regulating autophagy and mitochondrial dynamics.

## Introduction

Neurodegenerative diseases are neurological disorders and age-related diseases that generally progress slowly and worsen over time. These diseases are characterized by the loss of neuronal structure and function in the central nervous system (CNS) and peripheral nervous system (PNS)^1,2^, which leads to clinical features such as movement disorder, cognitive impairment, and behavioral impairment^3,4^. Wilson et al. recently reported the eight hallmarks of neurodegenerative disease consist of pathological protein aggregation, synaptic and neuronal network dysfunction, aberrant proteostasis, cytoskeletal abnormalities, altered energy homeostasis, DNA and RNA defects, inflammation, and neuronal cell death^5^. Notably, many of these characteristic features play a role and work together to facilitate neuronal cell death.

Glutamate is the most abundant excitatory neurotransmitter in the central nervous system (CNS), which plays a crucial role in synaptic communication and neuronal signaling. In conditions such as Alzheimer's, Parkinson's, and Huntington's diseases, there is an abnormal accumulation of glutamate in the brain's extracellular space^6–8^. The glutamate accumulation is the primary factor responsible for excessive ROS generation via the overactivation of glutamate receptor N-methyl-D-aspartate (NMDA), resulting in an excess calcium influx to the cells. Moreover, high extracellular glutamate can lead to glutathione depletion through cystine/glutamate antiporter (Xc). Subsequently, the accumulation of ROS leads to the deterioration of neuronal cells and triggers various types of cell death, such as apoptosis, necrosis, ferroptosis, and autophagy^9–11^. Among those, autophagy is a cellular process involved in the degradation and recycling of cellular components, including damaged proteins and organelles. In neurons, autophagy helps to remove misfolded proteins and damaged organelles, thus protecting neurons from cellular stress^12^. However, under specific abnormal circumstances, such as prolonged nutrient deprivation and chronic stress, autophagy can become excessive or uncontrolled, crossing a critical threshold where it triggers irreversible neuronal cell death. Moreover, autophagic cell death leads to the degradation of essential cellular components, especially mitochondria via mitophagy receptor PINK1 and parkin-mediated mitophagy or BNIP3 and NIX-dependent mitophagy^13,14^. The overactivation of the mitochondria degradation also process leads to an alteration in energy homeostasis. Consequently, inhibition of glutamate toxicity by targeting the excessive degradation process is regarded as a promising strategy for alleviating neurodegenerative disease.

The mammalian target of rapamycin complex 1 (mTORC1) is a critical regulator of cell growth, metabolism, and autophagy. It plays a central role in coordinating cellular responses to various stress conditions, including nutrient deprivation, energy depletion, and other forms of cellular stress. Under normal physiological conditions, mTORC1 fosters cell growth and protein synthesis. Simultaneously, it hampers autophagy by phosphorylating ULK1 (Unc-51-like autophagy activating kinase 1)^15^. However, mTORC1 activity is suppressed under stress conditions^16,17^. Thus, the mTORC1 regulation is important for maintaining cellular homeostasis and preventing uncontrolled autophagy.

Dietary supplements from plants containing phytochemicals such as carotenoids, flavonoids, saponins, and vitamins tend to prevent ROS or oxidative stress-related chronic conditions^18^. Interestingly, some antioxidant agents such as acetylcholinesterase inhibitor can improve learning and memory^19,20^. It is noteworthy that some natural bioactive compounds exhibit anti-aging properties and can restore mTORC1 activity^21^. *Albizia lebbeck,* a medicinal plant native to part of Southeast Asia and the Indian subcontinent, and belonging to the *Fabaceae* family, is recognized for its notable antioxidant, anti-inflammatory, and neuroprotective activities^22^. *A. lebbeck* contains various phytochemical constituent, which shows promise as a potential therapeutic agent for neurodegenerative diseases^23,24^. However, the neuroprotective effect and molecular mechanism of *A. lebbeck* extract and luteolin against glutamate-induced hippocampal cell death have not been elucidated. In this study, we identified that the leaf extract of *A. lebbeck* and luteolin have neuroprotective activities against glutamate-induced hippocampal neuronal cell death. Moreover, we unveiled a new mechanism by which luteolin hinders excessive mitophagy and autophagy through mTOR signaling (Supplementay Information).

## Results

### Glutamate-induced neuronal cell death

**Figure 1:**
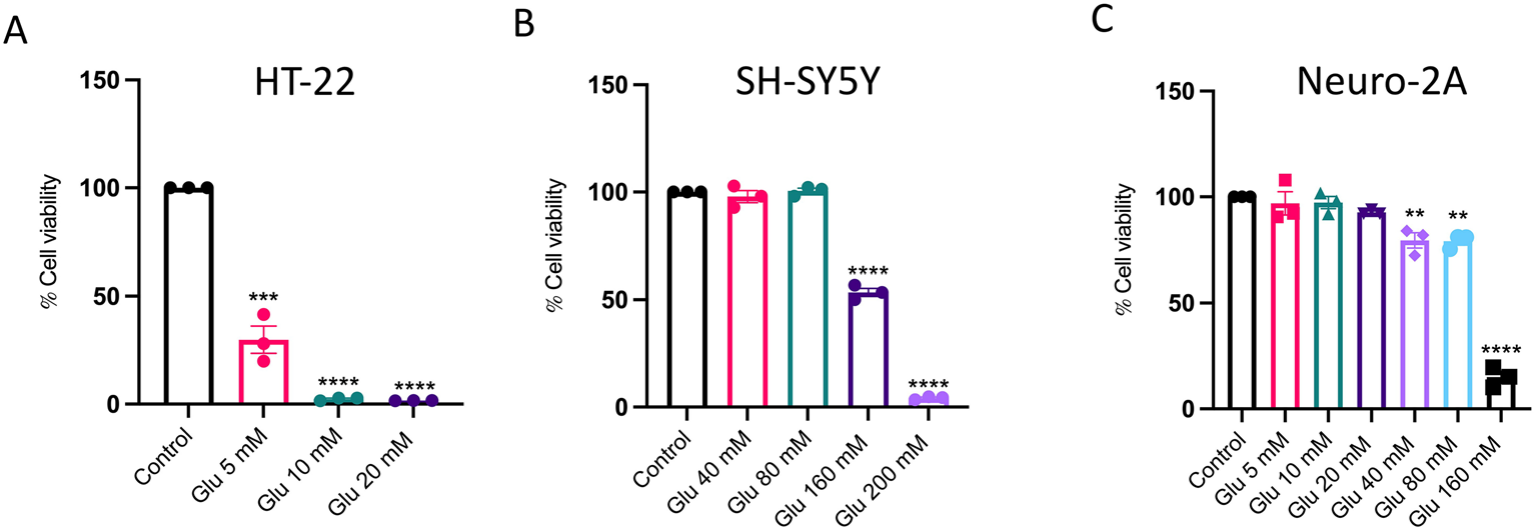
Glutamate-induced toxicity in different neuronal cells. The effect of glutamate-induced cytotoxicity in different types of neuronal cell lines was assessed by MTS assay (*n* = 3). HT-22 cells were treated with varying concentrations of glutamate, ranging from 5 to 20 mM. Similarly, SH-SY5Y mouse neuroblastoma cell line were treated with glutamate at 40–200 mM, and Neuro-2A mouse neuroblastoma cell line were treated with glutamate at 5–160 mM for 18 h. Bar graphs show the % cell viability of (**A**) HT-22 mouse hippocampal cell line, (**B**) SH-SY5Y mouse neuroblastoma cell line, and (**C**) Neuro-2A mouse neuroblastoma cell line. The data were collected from at least three independent experiments and the results were shown in mean $\pm$ SEM. **p-value < 0.01, ***p-value < 0.005, ****p-value < 0.001 compared with untreated control group.

Glutamate toxicity was examined in various neuronal cell lines to determine the optimal dose of glutamate and the most appropriate model. HT-22 mouse hippocampal, SH-SY5Y human neuroblastoma, and Neuro-2A mouse neuroblastoma cell lines were tested in our study and exposed to varying concentrations of glutamate. Cell viability was evaluated using the MTS assay, revealing that 5 mM of glutamate is the most optimal concentration for HT-22 cells. This concentration resulted in approximately 25% cell viability (Fig. 1A). Moreover, SH-SY5Y human neuroblastoma cells were treated with glutamate at various concentrations including 40, 80, 160, and 200 mM for 18 h. Glutamate toxicity in SH-SY5Y cells was observed to begin at a concentration of 160 mM, resulting in approximately 50% cell viability (Fig. 1B). In addition, the Neuro-2A mouse neuroblastoma cell line underwent treatment with varying concentrations of glutamate, revealing that the onset of glutamate toxicity occurs at 40 mM, leading to an approximately 80% cell viability (Fig. 1C). Given that among the tested neuronal cell lines, HT-22 cells displayed the highest sensitivity to glutamate toxicity, they were selected for the subsequent experiments.

### *A. lebbeck* leaf ethanol extracts (ALE) inhibit glutamate-induced HT-22 hippocampal neuronal cell death

**Figure 2:**
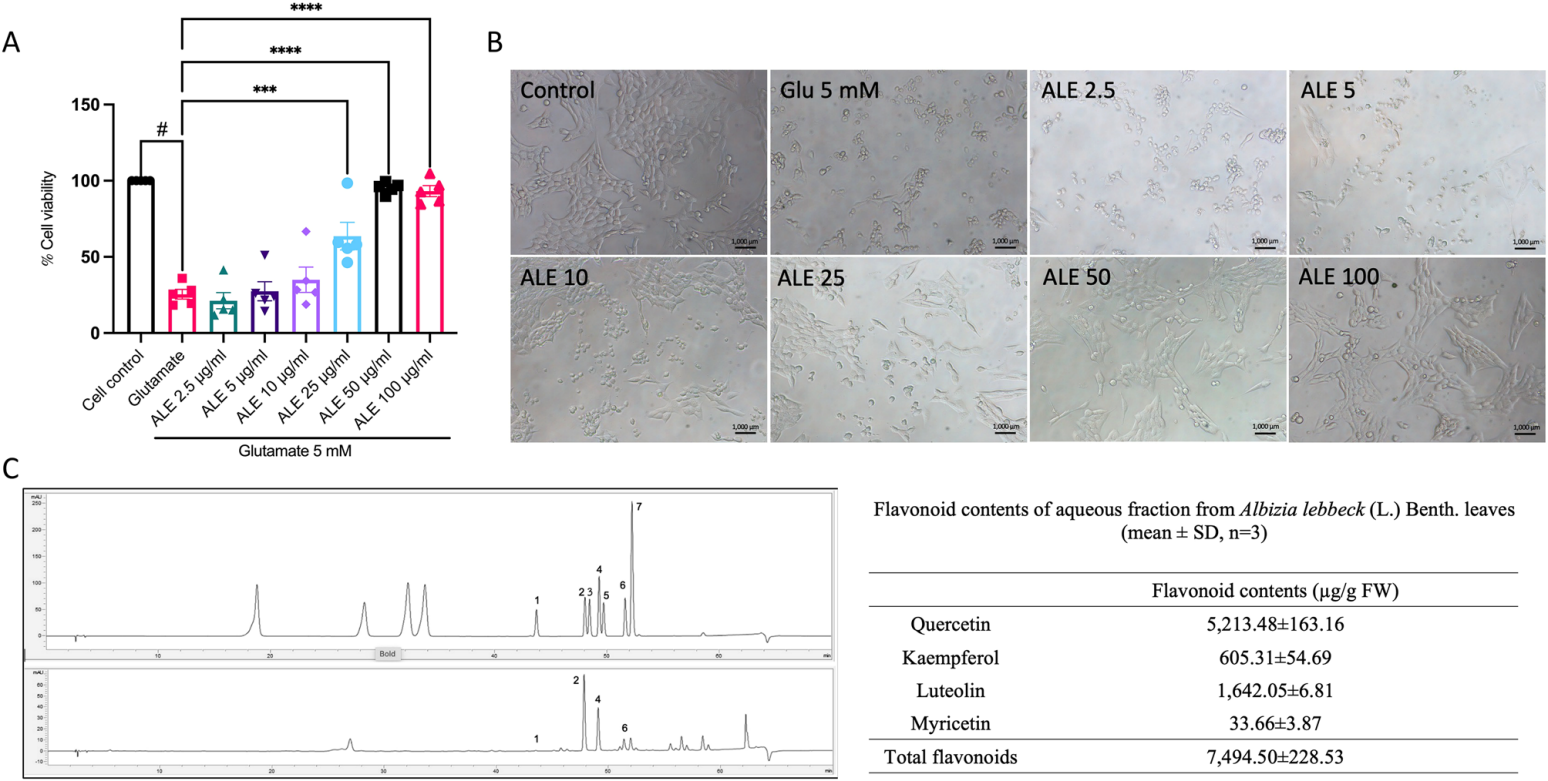
ALE inhibits glutamate-induced cytotoxicity in mouse HT-22 hippocampal cells. (**A**) The effect of ALE on glutamate-induced cytotoxicity in HT-22 was assessed by MTS assay (*n* = 5). HT-22 cells were pre-treated with ALE at different concentrations (2.5–100 µg/ml) for 24 h, followed by 5 mM glutamate for 18 h. Bar graphs show the % cell viability. (**B**) The morphology of HT-22 cells was visualized under the inverted light microscope (scale bar is 1000 µm). (**C**) HPLC chromatograms of flavonoid standards (Top) and *A. lebbeck* (L.) Benth. leaf (Bottom). (1 = myricetin; 2 = quercetin; 3 = naringenin; 4 = luteolin; 5 = hesperidin; 6 = kaempferol; 7 = apigenin). The data were collected from at least three independent experiments and the results were shown in mean $\pm$ SEM. ***p-value < 0.005, ****p-value < 0.001 compared with glutamate group. ^#^*p*-value < 0.05 compared with untreated control group.

Previous research has shown that extracts of *A. lebbeck* leaves can inhibit neuronal cell death^25^. In this study, we aimed to assess the inhibitory potential of ALE against glutamate-induced hippocampal neuronal cell death. Therefore, HT-22 cells were pre-treated with ALE at concentrations ranging from 2.5 to 100 µg/ml, followed by exposure to 5 mM of glutamate. The results revealed that 5 mM of glutamate led to approximately 25% cell viability in HT-22 cells. However, when cells were pre-treated with ALE, there was a significant dose-dependent increase in cell viability (Fig. 2A). Furthermore, upon examination under a light microscope, the treatment of cells with glutamate exhibited nuclear condensation and cell shrinkage. In contrast, those that were pre-treated with ALE retained their original cell morphology (Fig. 2B). To further investigate the flavonoid constituents of ALE, we employed the HPLC analysis (Fig. 2C). We found that ALE possesses a high flavonoid content, especially quercetin and luteolin. Therefore, we directed our attention to these two compounds for further investigation.

### Luteolin mitigates glutamate-induced cytotoxicity in HT-22 and reduces cellular stress

**Figure 3:**
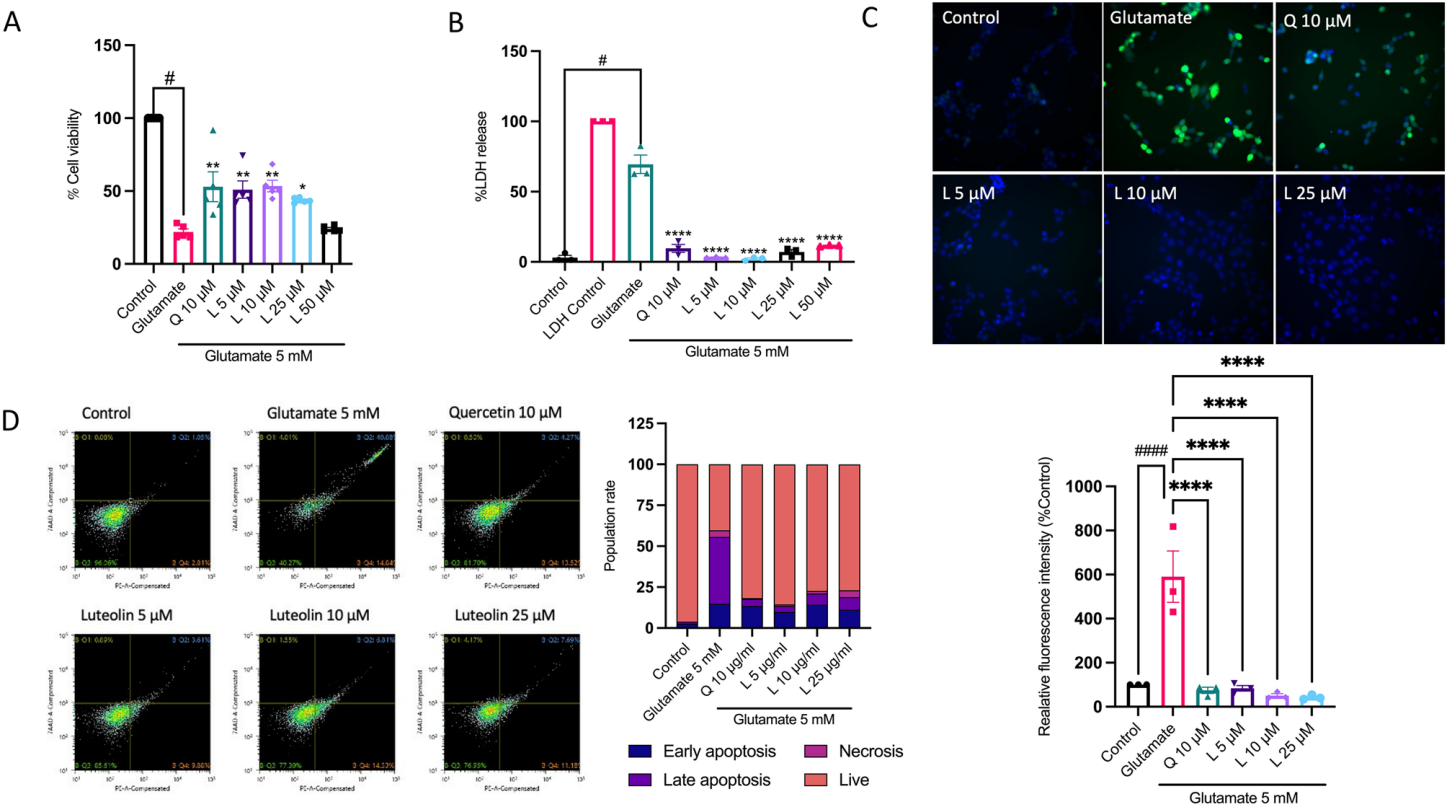
Luteolin inhibits glutamate-induced cytotoxicity in HT-22 cells. HT-22 cells were pre-treated with luteolin at different concentrations (5–50 µM) for 24 h and quercetin 10 µM was used as a positive control. Subsequently, the cells were exposed to 5 mM glutamate for 18 h. Bar graphs show (**A**) the % cell viability (*n* = 5) and (**B**) the % LDH release (*n* = 3). (**C**) The intracellular ROS was visualized under the CellInsight CX7 High-Content Screening (HCS) platform, the bottom bar graph shows the relative intracellular ROS level (*n* = 3). (**D**, left) The HT-22 cells were stained with PE-Annexin V/7-AAD probes, the numbers of cell deaths were analyzed via flow cytometry Q1: necrosis, Q2: late apoptosis, Q3: live, Q4: early apoptosis (*n* = 3). (**D**, right) The histogram represents the percentages of necrotic and apoptotic cells. The data were collected from at least three independent experiments and the results were shown in mean $\pm$ SEM. *p-value < 0.05, **p-value < 0.01, ****p-value < 0.001 compared with glutamate-treated group, ^#^*p*-value < 0.05, ^####^*p*-value < 0.001 compared with untreated control group. L:luteolin, Q:quercetin.

Considering our results, luteolin has been selected as the primary compound for investigating its neuroprotective effect against glutamate-induced HT-22 hippocampal neuronal death. To accomplish this, HT-22 cells were pre-treated with a range of luteolin concentrations (5–50 µM) for 24 h before exposing them to glutamate. We optimized the use of 10 µM quercetin (data not shown) as a positive control^26^. The cell viability assay result demonstrated that luteolin effectively restores HT-22 cell viability (Fig. 3A). Moreover, we used the LDH cytotoxicity assay to further support the cell viability data, showing that luteolin (5–50 µM) reduced glutamate toxicity in a dose-dependent manner (Fig. 3B). Glutamate is known to induce cytotoxicity in neuronal cells by promoting the production of ROS^27^. Therefore, we analyzed intracellular ROS levels using the H_2_DCF-DA probe to determine whether luteolin could alleviate glutamate-induced oxidative stress. Fluorescence intensity was measured in the CellInsight CX7 High-Content Screening (HCS) Platform and normalized with nuclear-specific staining (Hoechst 33342). Our findings revealed a notable elevation in intracellular ROS production due to glutamate exposure. Nevertheless, the pre-treatment of cells with luteolin and quercetin proficiently reinstated the levels of intracellular ROS accumulation (Fig. 3C). To assess the impact of luteolin on glutamate-induced cell death, we conducted an apoptosis assay using PE-Annexin V and 7AAD staining. The results demonstrated that treatment of HT-22 cells with 5 mM glutamate alone led to approximately 40% late apoptosis and 14% early apoptosis (Fig. 3D). However, pre-treatment with luteolin and quercetin prior to glutamate incubation significantly reduced glutamate-induced apoptosis, with the protective effect observed at a concentration of 5–25 µM and 10 µM respectively. Thus, cells pre-treated with luteolin showed a remarkable decrease in neuronal cell death induced by glutamate.

### Luteolin inhibits glutamate-induced mitochondrial dysfunction and restores mitochondrial content

**Figure 4:**
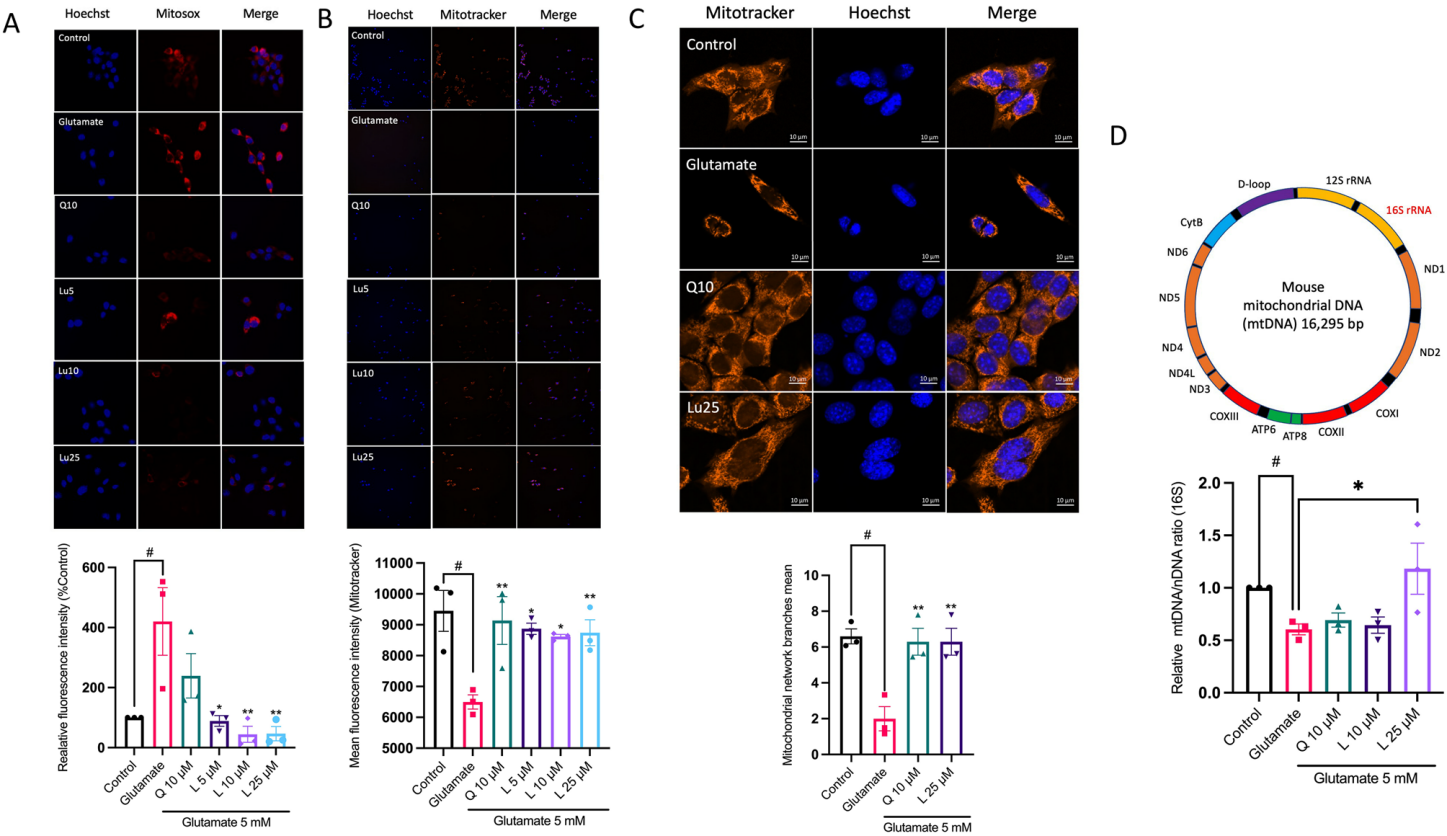
Luteolin reduces mitochondrial oxidative stress and restores mitochondrial function. (**A**) Representative the mitochondrial superoxide generation stained with mitosox indicator. HT-22 cells were pre-treated with luteolin at different concentrations (5–50 µM) for 24 h and quercetin 10 µM was used as a positive control, followed by 5 mM glutamate for 18 h. The bottom bar graph shows the relative mitochondria superoxide production (*n* = 3). (**B**) The mitochondria membrane potential was stained with mitotracker orange CMTMRos. The relative mean fluorescence staining was compared with control group (*n* = 3). (**C**) The mitochondria morphology was analyzed under a LSM 800 confocal microscope with 40X objective magnification (scale bar is 10 µm). The bottom bar graph shows the mitochondrial network branches mean analyzed with ImageJ Mitochondria Network Analysis plugin (MiNA) (*n* = 3). (**D**) The bar graph shows the relative mtDNA content (16S rRNA) / nuclear DNA ratio (*n* = 3). The data were collected from at least three independent experiments and the results were shown in mean $\pm$ SEM. *p-value < 0.05, **p-value < 0.01 compared with glutamate-treated group, ^#^*p*-value < 0.001 compared with untreated control group. L:luteolin, Q:quercetin.

Neurons have high energy demands to maintain their functions. Mitochondria are indeed the primary suppliers of adenosine triphosphate (ATP) in neurons and play a critical role in brain energy metabolism. Notably, glutamate excitotoxicity has been associated with mitochondria damage in neurons^28^. To assess the impact of luteolin on mitochondrial stress, the generation of mitochondria superoxide was evaluated utilizing the mitosox probe. The fluorescence intensity was then monitored using the CellInsight CX7 high content screening (HCS) Platform, with subsequent normalization with Hoechst 33342. Our result revealed that glutamate induced an increase in mitochondria superoxide production. However, pre-treatment of cells with luteolin (5–25 µM) and quercetin (10 µM) significantly restored the mitochondria superoxide level (Fig. 4A). Furthermore, the mitochondria membrane potential, which is susceptible to oxidative stress, was evaluated by staining with mitotracker orange CMTMRos (Fig. 4B). The control group (untreated group) showed a high fluorescent intensity, indicating a robust mitochondrial membrane potential. In contrast, the glutamate treatment group displayed a significant reduction in fluorescent intensity, demonstrating a loss of mitochondrial membrane potential. However, pre-treatment with luteolin and quercetin protected the mitochondrial membrane potential and restored it. Quantification of fluorescence intensity for mitotracker orange CMTMRos confirmed that glutamate significantly decreased the fluorescence intensity compared to the control group (Fig. 4B, bottom). Additionally, we used mitotracker orange CMTMRos staining to assess mitochondria morphology under the confocal microscope. The mitochondria network analysis plugin (MiNA) was used to analyze mitochondria morphology. Our results showed that glutamate could cause mitochondria fragmentation and decrease the branching network. However, pre-treatment of cells with luteolin and quercetin prior to glutamate exposure could maintain the mitochondria morphology and increase the branching network (Fig. 4C). Moreover, we investigated the mitochondrial content using the stable mtDNA fraction, 16S rRNA, comparing it with nuclear DNA (HKII). We found that the glutamate treatment group had a decrease in mtDNA/nDNA ratio, indicating the loss of mitochondria content (Fig. 4D). Conversely, the luteolin pre-treatment group had an increase in mtDNA/nDNA ratio, indicating an increase in mitochondria content. These findings suggest that luteolin has a positive impact on mitochondrial health and may protect against glutamate-induced mitochondrial stress.

### Luteolin inhibits the excessive mitochondria degradation process and autophagy-mediated neuronal cell death

**Figure 5:**
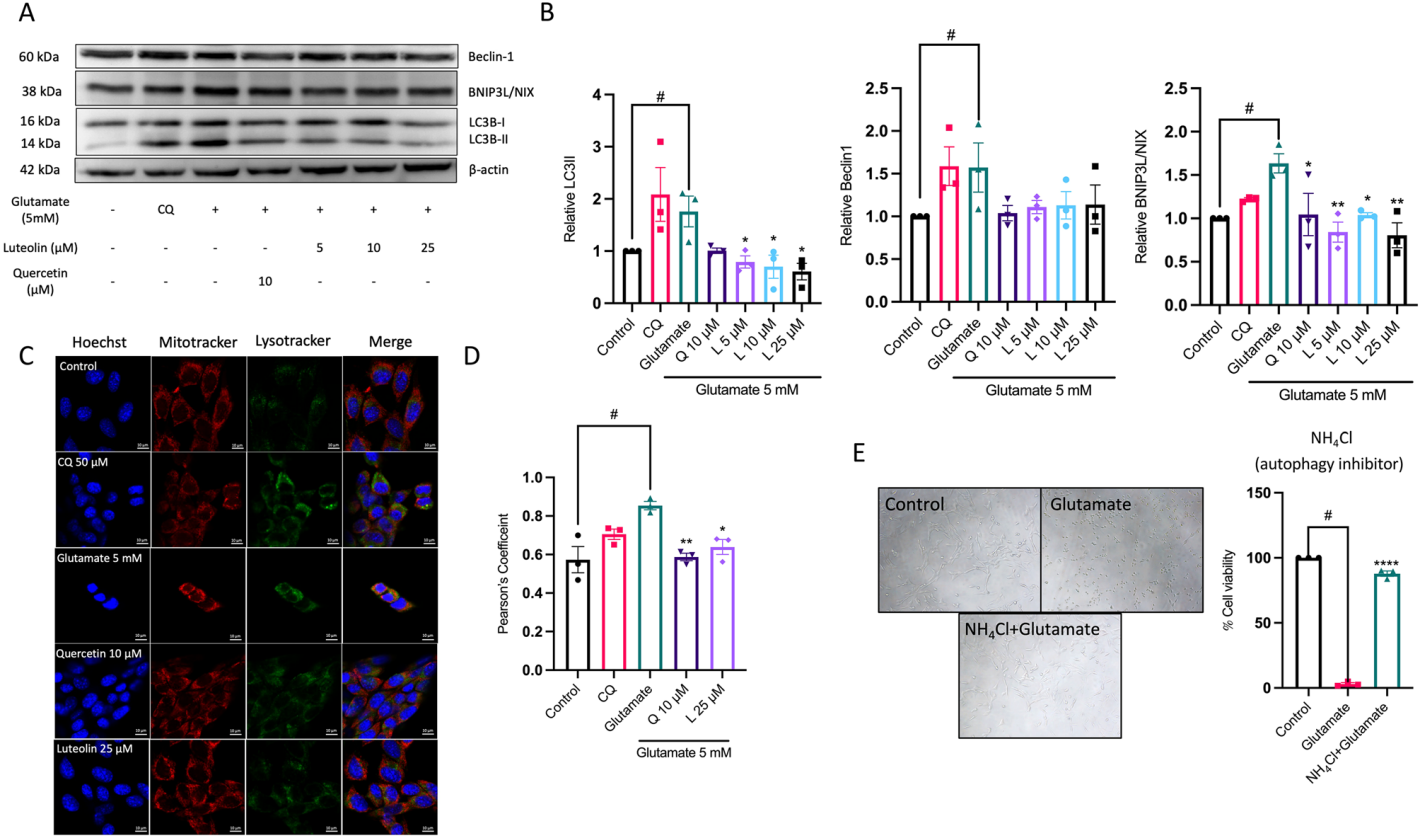
Luteolin inhibits glutamate-induced excessive autophagy and mitophagy activation. HT-22 cells were pre-treated with luteolin at different concentrations (5–50 µM) for 24 h and 10 µM quercetin was used as a positive control, followed by 5 mM glutamate for 18 h. Chloroquine (CQ, 50 µM) served as the positive control for autophagy induction. (**A**) The protein expression level of LC3B, Beclin-1 (autophagy) and BNIP3L/NIX (mitophagy) were analyzed by Western blot, and β-actin served as the loading control. (**B**) Relative protein levels of LC3B, Beclin-1 (autophagy) and BNIP3L/NIX (mitophagy) were quantified by densitometry and the mean data from at least three independent experiments were normalized to the results (*n* = 3). (**C**) The co-localization of lysosome and mitochondria. HT-22 cells were stained with the mitochondria (Mitotracker: red) Lysosome (Lysotracker: green) and nucleus (Hoechst: blue). They were observed under the confocal laser scanning microscope with 40X objective magnification (scalebar is 10 µm). (**D**) The bar graph of co-localization was considered with Pearson’s correlation coefficient analyzed using ImageJ JACoP plugin. Data represent the means ± SEM and represent averages of results from at least 50 cells (*n* = 3). (**E**) Autophagy inhibitor, ammonium chloride (NH_4_Cl), inhibits glutamate-induced HT-22 cell death. The morphology of HT-22 cells was visualized under the inverted light microscope. A bar graph shows the MTS cell viability results (*n* = 3). The data represent the means ± SEM collected from at least three independent experiments and. *p value < 0.05, **p value < 0.01, ****p value < 0.001 compared with only glutamate-treated group, ^#^*p* value < 0.05 compared with untreated control group. CQ:chloroquine, L:luteolin, Q:quercetin.

To gain insight into the mechanism underlying the glutamate-induced reduction in mitochondria content, we investigated cellular degradation processes. This involved examining protein expression levels of autophagy markers, namely LC3B and Beclin-1 using Western blot analysis. In line with a previous study^29^, we employed chloroquine at a concentration of 50 µM to stimulate autophagy in HT-22 cells, serving as a positive control for verifying autophagy activation. Our findings demonstrate that both the glutamate-treated group and the chloroquine-treated group exhibited elevation in protein levels of LC3B-II (autophagosome marker) and Beclin-1, compared to the untreated group. Interestingly, both luteolin and quercetin exhibited inhibitory effects on LC3B conversion and led to a decrease in Beclin-1 protein expression levels as compared to the glutamate-treated group (Fig. 5A,B). In addition, we investigated the protein expression of the stress-sensitive mitophagy receptor BNIP3L/NIX. Notably, the glutamate treatment group showed an increase in BNIP3L/NIX protein expression, suggesting an over-activation of the mitochondria degradation process by glutamate. In contrast, both luteolin and quercetin treatments significantly decrease the level of BNIP3L/NIX protein expression compared to the glutamate-treated group (Fig. 5A,B). The co-localization of lysosomes and mitochondria was used to clarify the glutamate-stimulated excessive mitophagy. The results revealed that the chloroquine and glutamate-treated group exhibited an increase in lysosomal fluorescence intensity, suggesting lysosomal activation. In contrast, the cells treated with luteolin and quercetin showed a decrease in lysosomal fluorescence intensity, suggesting a potential suppression of lysosomal activity (Fig. 5C). Moreover, we computed the Pearson's correlation coefficient to evaluate co-localization. Co-localization was observed in the group receiving glutamate treatment, while it decreased in the groups treated with luteolin and quercetin (Fig. 5D). These results suggest that glutamate can lead to the overactivation of the mitophagy process, but this effect can be inhibited by luteolin. Finally, we investigated whether overactivation of the mitophagy process results in HT-22 cell death by inhibiting autophagolysosomal fusion with ammonium chloride (NH_4_Cl) and assessing cell viability with the MTS assay. Our findings indicated that NH_4_Cl increased the cell viability compared to the glutamate treatment group, suggesting that the overactivation of the mitophagy process is associated with HT-22 cell death (Fig. 5E).

### Luteolin triggers the activation of mTORC1 to prevent glutamate-induced autophagy-mediated cell death.

**Figure 6:**
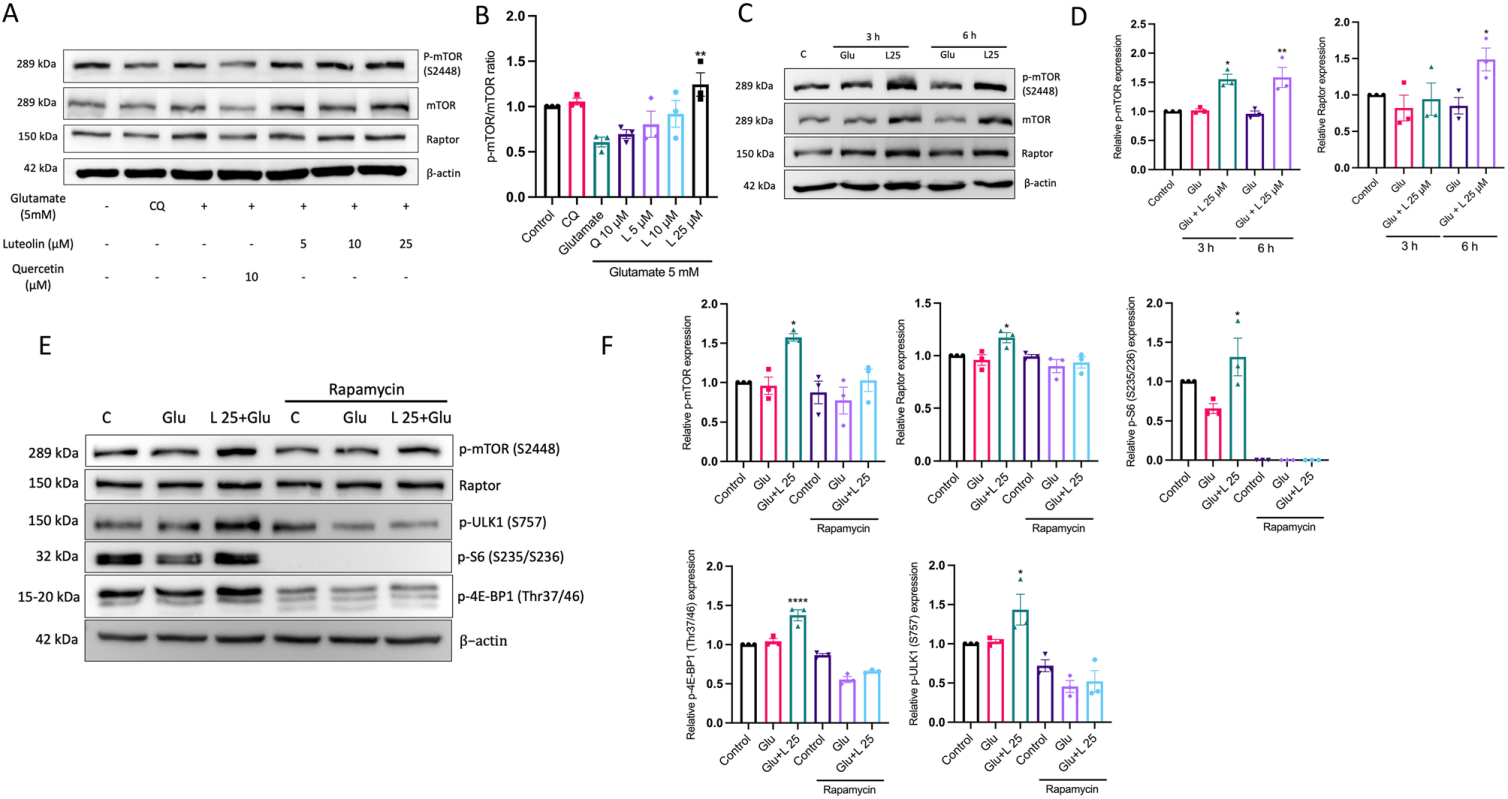
Luteolin increases the mTORC1 activation. HT-22 cells were pre-treated with luteolin at 5–25 µM for 24 h, followed by 5 mM glutamate for 18 h. (**A**) The dose-dependent mTORC1 protein expression. The protein expression level of p-mTOR S2448 (mTOR activation) and Raptor (mTORC1 complex) were analyzed by Western blot, and β-actin served as the loading control. (**B**) Relative protein levels of p-mTOR S2448 (mTOR activation) and Raptor (mTORC1 complex) were quantified by densitometry and the mean data from at least three independent experiments were normalized to the results (*n* = 3). (**C**) The time dependent mTORC1 protein expression. The protein expression level of p-mTOR S2448 (mTOR activation) and Raptor (mTORC1 complex) were analyzed at 3 h or 6 h of glutamate induction, and β-actin served as the loading control. (**D**) Relative protein levels of p-mTOR S2448 (mTOR activation) and Raptor (mTORC1 complex) (*n* = 3). (**E**) Representative immunoblotting of mTORC1 downstream target p-S6 (S235/236), p-4E-BP1 (Thr37/46), and p-ULK1 (S757). (**F**) Relative protein levels of p-mTOR S2448, Raptor, p-S6 (S235/236), p-4E-BP1 (Thr37/46), and p-ULK1 (S757) after rapamycin treatment (*n* = 3). The Western blot was quantified using NIH Imaging J. The data represent the means ± SEM collected from at least three independent experiments and. *p value < 0.05, **p value < 0.01, ****p value < 0.001 compared with the glutamate treatment group. C:control, Glu:glutamate, L:luteolin, Q:quercetin.

The mammalian target of rapamycin complex 1 (mTORC1) regulates autophagy but is often inhibited during stress conditions^16,30^. To explore this further, we conducted an experiment on HT-22 cells. The cells were pre-treated with luteolin at various concentrations (5–25 µM) and then exposed to glutamate for 18 h. We evaluated the mTOR phosphorylation level at S2448. Phosphorylation at this particular site is thought to regulate the function of mTORC1 and its ability to inhibit autophagy^31^. Our result revealed that the mTOR phosphorylation at S2448 decreased in the glutamate treatment group. In contrast, luteolin increases mTOR phosphorylation at S2448 in a dose-dependent manner (Fig. 6A,B). However, quercetin did not restore the mTOR phosphorylation levels, suggesting that quercetin may inhibit autophagy activation through other pathways. We also measured the protein level of a biomarker of the mTORC1 complex (Raptor); however, no significant change in the Raptor protein was observed after 18 h of glutamate induction (Fig. 6A). To assess the impact of luteolin on mTORC1 activation, HT-22 cells were pre-treated with 25 µM of luteolin and then exposed them to glutamate for 3 and 6 h. Our result revealed that the mTOR phosphorylation at S2448 significantly increased in the luteolin-treated group at 3 and 6 h. Interestingly, the increase in Raptor protein level was observed at 6 h of glutamate exposure (Fig. 6C,D). Furthermore, to confirm that luteolin triggers the activation of mTORC1 under glutamate-induced neuronal cell death, we used rapamycin, which decreases mTOR activity without affecting its abundance. Rapamycin was pre-treated for 1 h before glutamate induction for 6 h. The results showed that luteolin increases the protein expression of p-mTOR (S2448), Raptor, and the levels of mTOR downstream targets, such as p-S6 (S235/236), p-4E-BP1 (Thr37/46), and p-ULK1 (S757). Moreover, p-S6 (S235/236) was decreased in the glutamate-treated group, indicating the inhibition of mTORC1 activity (Fig. 6E,F). Whereas rapamycin treatment inhibited the mTORC1 activation in the luteolin treatment group. Thus, these data demonstrated that glutamate suppressed mTORC1 activity, while luteolin stimulated the mTORC1 activity.

### Luteolin decreases the mitophagy receptor and mTORC1 downstream target UVRAG mRNA expression

**Figure 7:**
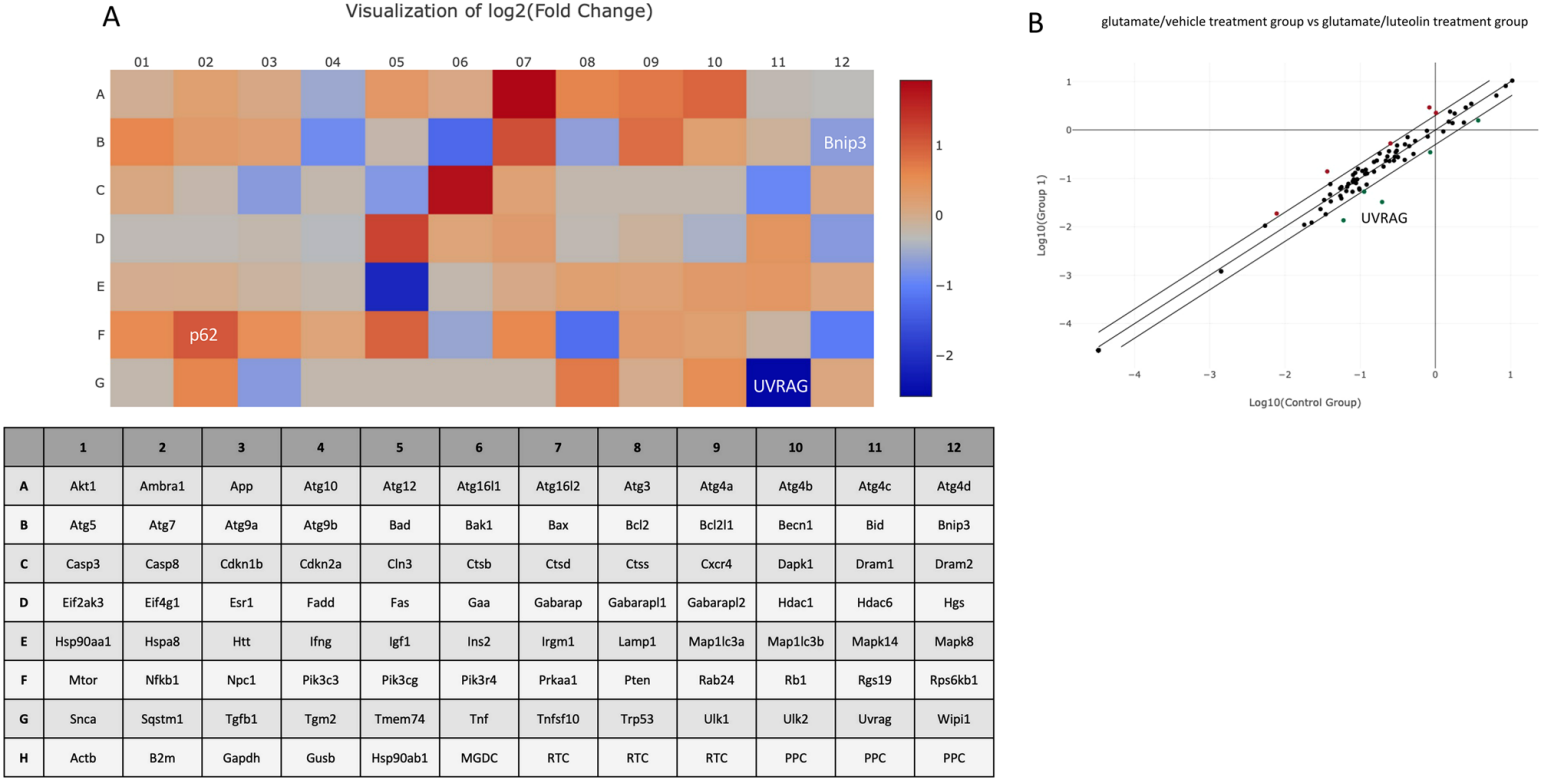
Luteolin inhibits autophagy-related mRNA expression. Luteolin decreases the Bnip3 (Mitophagy receptor) and UVRAG (mTORC1 downstream target) mRNA expression and increases p62 (Autophagy substrate) mRNA expression. HT-22 cells were pre-treated with luteolin at 25 µM for 24 h, followed by 5 mM glutamate for 12 h. (**A**) The heatmap results of mouse autophagy PCR array of glutamate-treated vs luteolin + glutamate treated. Upregulation: red, Down regulation: blue. (**B**) The scatter plot of mRNA expression with fold regulation = 2, upregulation: red, down regulation: green.

To investigate the impact of luteolin on mRNA expression related to autophagy, we conducted a comparative analysis of 84 autophagy-related genes between the glutamate/vehicle treatment group and the glutamate/luteolin treatment group using the RT2 profiler mouse autophagy PCR array. As shown in Fig. 7A, luteolin was found to increase the mRNA expression of the autophagy substrate p62. Furthermore, luteolin induced a reduction in the mRNA expression of BNIP3, a factor implicated in promoting mitophagy via BNIP3L/NIX. Interestingly, luteolin also contributed to a reduction exceeding twofold in the mRNA expression of the Ultraviolet irradiation resistance-associated gene (UVRAG), a crucial protein in autophagosome formation (Fig. 7B). Since the previous study reported the negative regulation between mTORC1 and UVRAG^32^, PCR array results are supportive evidence that luteolin inhibits autophagy activation through increasing mTORC1 activity.

## Discussion

Neuronal cell death is a complex and multifactorial process that plays a critical role in various neurological disorders and neurodegenerative diseases. One significant factor in this cell death is the oxidative stress caused by excessive glutamate^8,33–36^. Glutamate is an excitatory neurotransmitter that plays a crucial role in normal brain function; however, excessive release or impaired clearance of glutamate can lead to excitotoxicity, triggering a cascade of events that ultimately results in neuronal cell death. Glutamate toxicity has been reported in many cases of neurodegenerative disorders and accelerates the pathophysiology of neurodegenerative diseases^37–39^.

In this study, we present the protective effects of *A. lebbeck* leaf extract (ALE) and its primary active component luteolin, against oxidative stress caused by glutamate in mouse hippocampal neuronal cells. To start, we evaluated three distinct brain cell lines (mouse hippocampus, mouse neuroblastoma, and human neuroblastoma) and ultimately chose HT-22 mouse hippocampal cells because of their increased susceptibility to glutamate-induced toxicity (Fig. 1). This sensitivity is probably due to the lack of N-methyl-D-aspartate (NMDA) receptor and the mechanism is undergoing through cysteine glutamate antiporter, which consequently triggers a reduction in glutathione level^11^. Of importance, HT-22 cells have shown the presence of Alzheimer's disease-specific markers under glutamate toxicity conditions^40^ and it is worth noting that glutamate-induced oxidative toxicity contributes to 50% of neuronal cell fatality^41^. Thus, HT-22 cells can serve as a valuable model system for assessing and testing novel agents with potential anti-neurodegenerative disease properties.

Our study demonstrates that ALE enhances the survival of neuronal cells against glutamate-induced neuronal cell death. ALE contains a substantial number of flavonoids, with quercetin and luteolin as major phytochemical components (Fig. 2C). Notably, quercetin is the predominant compound within ALE and has previously reported neuroprotective effects against glutamate oxidative toxicity in HT-22 cells^26,42^. On the other hand, luteolin is the second major phytochemical which is a natural flavonoid compound found in various fruits, vegetables, and herbs. It is also reported in several studies that luteolin exhibits neuroprotective effects by acting as an antioxidant and anti-inflammatory agent^24,43^. Despite this, the specific neuroprotective mechanisms of luteolin against glutamate toxicity have not been fully understood. Our findings reveal that luteolin effectively prevents glutamate-induced neuronal apoptosis and various other forms of cell death, while also diminishing the accumulation of intracellular ROS (Fig. 3).

Mitochondria are the most abundant in neurons and essential for neuronal function. During glutamate excitotoxicity, mitochondria are involved in the intrinsic apoptotic pathway by releasing pro-apoptotic factors such as cytochrome c and apoptosis-inducing factor (AIF) into the cytoplasm, triggering a cascade of events that ultimately lead to cell death^44^. This situation compromises vital aspects like maintaining the potential of mitochondrial membranes and the characteristic branching of mitochondria, culminating in the accumulation of impaired mitochondria within neuronal cells^45,46^. Apparently, luteolin intervenes in this process, effectively curbing mitochondrial dysfunction and even restoring the count of functional mitochondria, as shown in Fig. 4. These outcomes might be attributed to luteolin's antioxidant activity, which recent studies have linked to hindering elevated calcium levels and addressing mitochondrial dysfunction^47^. Interestingly, previous in vivo studies revealed that luteolin ameliorates Alzheimer’s disease mice via inhibited endoplasmic reticulum stress and inhibited Aβ-induced mitochondrial dysfunction and neuronal apoptosis^48,49^. Additionally, in a stroke model, luteolin mitigated CA1 hippocampal damage by reducing glial cell activation and suppressing autophagy in MCAO/R-treated rats. Furthermore, it decreased mitochondrial vacuolization^50^. These results suggest that luteolin serves as a neuroprotective compound in conditions characterized by mitochondrial dysfunction and oxidative stress. In parallel, damaged mitochondria and other cellular components can be eliminated and renewed through the autophagy and mitophagy processes^51^. Intriguingly, prolonged periods of stress can provoke an excessive autophagy and mitophagy response, eventually leading to the demise of neuronal cells. These occurrences have been documented in a range of instances involving neuronal cell death^52–54^.

BNIP3L (BCL2/adenovirus E1B 19kDa interacting protein 3-like), also known as NIX (Nip3-like protein X) localizes at mitochondria outer membrane which serves as a stress sensing protein and induction of cell death. Moreover, BNIP3L/NIX acts as a mitophagy receptor that interacts with LC3 through its LIR (LC3-interacting region) domain. This interaction serves to attract LC3 family proteins to impaired mitochondria^55^. Our findings firstly demonstrate that there is an increased expression of BNIP3L/NIX and the presence of the autophagy marker LC3 following exposure to glutamate. Furthermore, we observed the activation of lysosomes along with their colocalization with mitochondria, suggesting the initiation of cellular degradation and mitophagy processes (Fig. 5). These observations align with an earlier study, indicating that glutamate prompts the activation of autophagy^56^.

Regarding the impact of glutamate-induced toxicity on HT-22 cells, we also observed increased levels of mitophagy. Damaged mitochondria are identified with the aid of diverse mitophagy-related proteins and PINK1 augments the mitophagy signaling by generating phosphorylated ubiquitin, facilitating the connection of autophagosomal components like P62 and LC3 to envelop the targeted mitochondria^57^. Alternatively, both BNIP3L/NIX and BNIP3 respond to cellular stress signals, orchestrating the recruitment of autophagosomes. Moreover, BNIP3L/NIX has been documented to trigger the disruption of mitochondrial transmembrane potential^58^. As a result, these pathways synergistically contribute to the excessive degradation of mitochondria. Luteolin intervenes in these processes, reinstating the integrity of mitochondria and counteracting mitochondrial stress. This intervention leads to a decline in the protein expression of mitophagy receptors (BNIP3L/NIX) and autophagy markers (LC3). Notably, luteolin has been found to inhibit autophagy activation induced by ovalbumin (OVA), acting via the PI3K/Akt/mTOR pathway, and suppressing the Beclin-1 complex^59^.

A pivotal regulatory protein mTOR governs an array of cellular processes encompassing cell growth, proliferation, and autophagy. Particularly in neurons, mTOR exerts control over neuronal development, function, and survival. Activation of mTORC1 yields the ability to temper excessive autophagy, while also influencing protein synthesis and remodeling of the cytoskeleton. This orchestration facilitates neuronal expansion, dendritic and axonal branching^60,61^. Perturbation of mTOR signaling has been linked to neurodegenerative pathways^30,62^. Our study reveals that prolonged exposure to glutamate diminishes the phosphorylation of mTOR at the s2448 site, a key regulator of mTOR activity (Fig. 6A). During instances of cellular stress, the functionality of mTORC1 diminishes due to a lack of energy and inadequate energy availability adversely impacts mTORC1's operational capacity, given its dependence on ample ATP levels. Intriguingly, our findings indicate that luteolin restores mitochondrial function, while concurrently upregulating mTOR phosphorylation. This phosphorylation occurs at the S2448 position of mTOR and is present within both mTORC1 and mTORC2 complexes.

The impact of luteolin on mTORC1 activation was confirmed using rapamycin treatment, as shown in Fig. 6. Luteolin prompts an increase in the protein expression of key downstream targets of mTORC1, such as p-S6 (S235/236), p-4E-BP1 (Thr37/46), and p-ULK1 (S757). In contrast, rapamycin treatment suppresses mTORC1 activation in the luteolin-treated group. A noteworthy protein, ULK1, plays a crucial role in initiating both autophagy and mitophagy processes. It collaborates with other autophagy-related proteins like ATG13, FIP200, and ATG101 to initiate the assembly of autophagosomes^61^. Furthermore, ULK1's activation at the mitochondria signals the start of engulfing damaged mitochondria by autophagosomes. Our data indicated that luteolin increased ULK1 phosphorylation at the S757 site (inactive form), thereby hindering the initiation of autophagy. Interestingly, luteolin also increases the expression of UVRAG mRNA, as shown in Fig. 7. UVRAG interacts with Beclin-1 and class III phosphatidylinositol 3-kinase (PI3K) complexes, which support the formation of precursors for autophagosomes. Based on array data, luteolin steps into reducing excessive autophagy and mitophagy triggered by glutamate through mTORC1 activation. This subsequently inhibits the ULK1-mediated assembly of autophagosomes and suppresses the expression of UVRAG, thereby curbing the initiation of autophagosome formation. In this investigation, luteolin demonstrated the ability to inhibit autophagy and mitophagy through mechanisms involving the suppression of reactive oxygen species (ROS) accumulation, restoration of mitochondrial function, and activation of mTORC1. Nevertheless, it is noted that luteolin exhibits divergent effects across various cellular contexts. While luteolin presents potential in cancer therapy through its modulation of autophagy, its impact on autophagic processes is contingent upon the specific autophagic activity within each cell type^63,64^. Moreover, the effect depends on the concentration of luteolin.

## Conclusion

**Figure 8:**
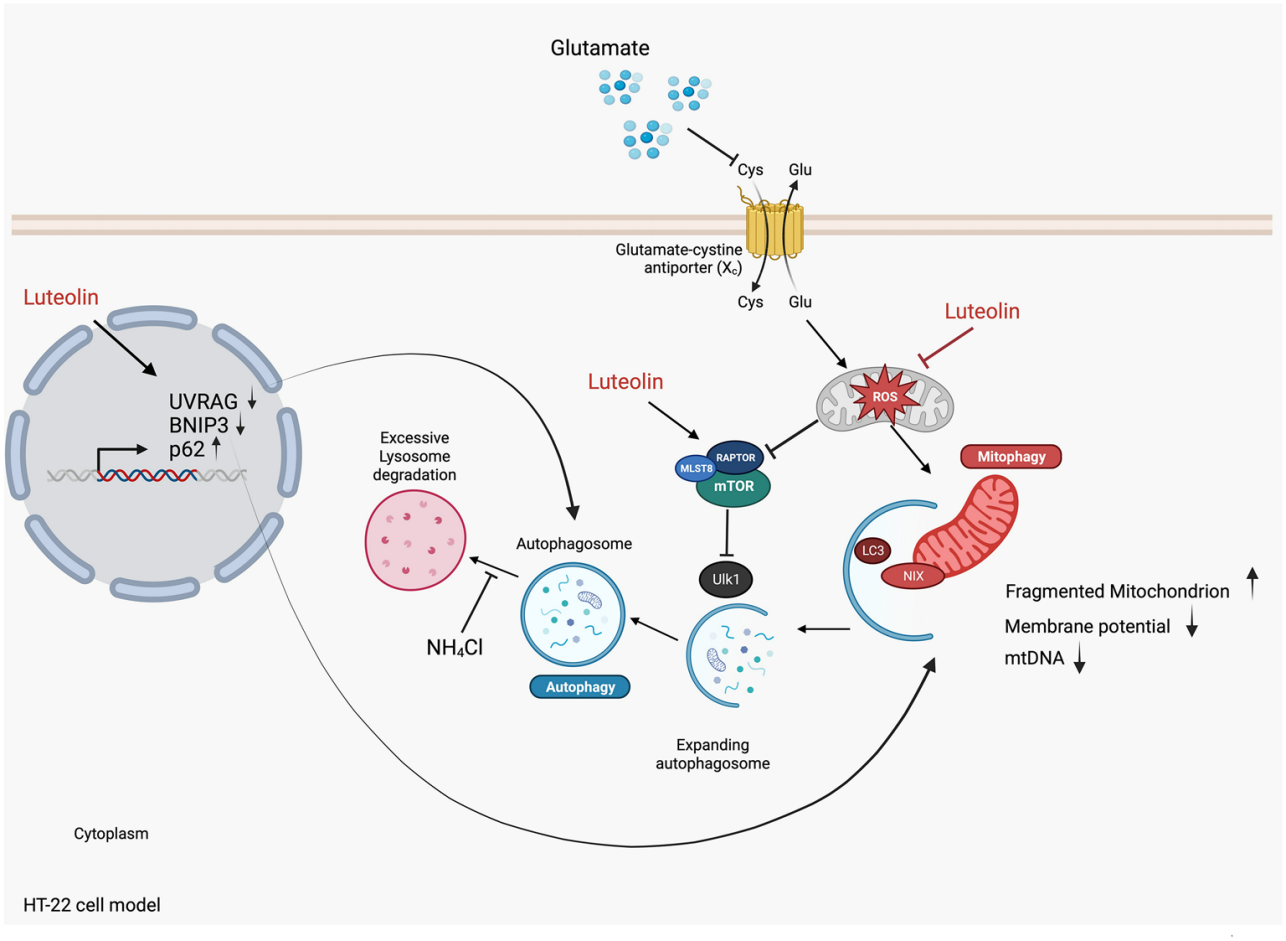
A proposed mechanism of luteolin against glutamate-induced neuronal death through autophagy-mediated neuronal cell death. Elevated glutamate levels within neurons cause an increase in mitochondrial ROS. These heightened ROS levels can damage mitochondria and trigger an excessive and specific process of removing damaged mitochondria, known as mitophagy, facilitated by BNIP3L/NIX. This sequence of events ultimately leads to the death of neurons. On the other hand, luteolin directly counteracts ROS, rescues mitochondrial health, and enhances the expression of mTORC1. Moreover, luteolin decreases autophagy-related gene expression (BNIP3 and UVRAG) and increases p62 gene expression. Furthermore, luteolin can reduce the initiation of mitophagy, resulting in a reduction of neuronal cell death. This figure was created with BioRender.com.

In this study, we unveil luteolin's ability to prevent glutamate-induced neuronal apoptosis and reduce ROS accumulation. Remarkably, luteolin restores mitochondrial function, mitigates mitochondrial dysfunction, and curtails excessive autophagy and mitophagy as shown in Fig. 8. This protective role might stem from its antioxidant attributes. However, further investigations are required to examine both animal and clinical studies for more understanding and clarifying the neuroprotective effects and deep mechanisms of this *A. lebbeck* leaf. Overall, our findings provide valuable insights into luteolin's potential as a therapeutic agent against neurodegenerative diseases.

## Materials and methods

### Plant collection and extraction

The *A. lebbeck (L.)* Benth. leaves were collected during February to March from the Srinakarind Dam area in Kanchanaburi province, Thailand. The permission for plant sample collecting was obtained from the Plant Genetic Conservation Project initiated by the Royal Patronage of Her Royal Highness Princess Maha Chakri Sirindhorn. All protocols concerning plant access and collecting were in accordance with Thai national regulations, notably the Plant Variety Protection Act (1999), obtaining appropriate permits and maintaining ethical academic standards throughout the process. This plant was identified and confirmed as a scientific name by Assistant Professor Dr. Thaya Jenjittikul, Department of Plant Science, Faculty of Science, Mahidol University, Thailand. A voucher specimen was deposited at Suan Luang Rama IX Herbarium, Bangkok, Thailand (No.9429).

The edible part of the *A. lebbeck* leaves was separated and washed with deionized water according to the procedure described by Phoraksa and colleagues^25^. Briefly, *A. lebbeck* leaves were boiled for 2 min, followed by homogenization using an electric blender. Subsequently, the homogenized leaf mixture underwent lyophilization using a freeze dryer. The resultant *A. lebbeck* powder was then subjected to ethanol extraction. For the extraction process, 2 g of the sample was dissolved in 30 ml of each solvent. The mixture underwent sonication in a sonicator bath for 10 min, followed by centrifugation at 3,000 rpm. for 10 min. The resulting supernatant was collected in amber glass bottles. Subsequently, the extract was dried using a rotary evaporator and nitrogen blower. The stock solution was prepared by dissolving in DMSO and kept at − 20 °C. The study was conducted in accordance with relevant guidelines and legislation.

### Determination of flavonoid contents

The modified method from Dawilai et al. was used to assess the flavonoid contents^65^. First, the ALE sample was subjected to acid methanol hydrolysis to obtain the aglycone form. Subsequently, the sample was boiled with a mixture of 62.5% (v/v) methanol, t-butyl hydroquinone (0.5 g/L), and 6 N hydrochloric acid for 2 h. Afterward, the sample was cooled on ice for 5 min, followed by the addition of 0.1% (w/v) ascorbic acid. Next, the sample was sonicated for 5 min and then filtered using a 0.2 µm PTFE syringe filter. Samples were analyzed with HPLC (Agilent 1260 Series liquid chromatograph, USA) using a ZORBAX Eclipse XDB-C18 column (4.6 × 150 mm). The mobile phase contains 0.1% trifluoroacetic acid (TFA) in water and 0.1% TFA in methanol. Quantification of flavonoid contents was accomplished by comparing retention times and spectral absorption with standard compounds (namely myricetin, quercetin, kaempferol, luteolin, apigenin, naringenin, and hesperidin). The results are presented as µg/g FW.

### Antibodies and reagents

Antibody: LC3B (D11 Cell Signaling Technology, Danvers, MA, USA), BNIP3L/NIX (D4R4B Cell Signaling Technology, Danvers, MA, USA), Beclin-1 (D40C5 Cell Signaling Technology, Danvers, MA, USA), Phospho-mTOR (ser2448) (D9C2 Cell Signaling Technology, Danvers, MA, USA), mTOR (7C10 Cell Signaling Technology, Danvers, MA, USA), Raptor (24C12 Cell Signaling Technology, Danvers, MA, USA), Phospho-S6 Ribosomal Protein (Ser235/236 Cell Signaling Technology, Danvers, MA, USA), Phospho-4E-BP1 (Thr37/46) (236B4 Cell Signaling Technology, Danvers, MA, USA) mouse anti-β-actin (C4) HRP (#SC47778 Santa Cruz Biotechnology, Dallas, TX, USA) and goat anti-rabbit IgG (H + L) secondary antibody, HRP conjugate (#31,460 Invitrogen, Carlsbad, CA, USA). Dimethyl sulfoxide (DMSO) (DR1022) was purchased from Biosesang (Gyeonggi, South Korea). Fetal bovine serum (FBS), Dulbecco's Modified Eagle Medium (DMEM), phosphate-buffered saline (PBS), penicillin–streptomycin, SuperSignal™ West Femto maximum sensitivity substrate, SuperSignal™ West Pico PLUS chemiluminescent substrate, MitoTracker™ Orange CM-H2TMRos (Cat. #M7510), LysoTracker™ Deep Red (Cat. #L12492), MitoSOX™ mitochondrial superoxide indicators (Cat. #M36008) and Pierce™ BCA protein assay kit were purchased from Thermo Scientific (Waltham, MA, USA). The CellTiter 96® AQueous One Solution Cell Proliferation Assay (MTS) and CytoTox 96® Non-Radioactive Cytotoxicity Assay were purchased from Promega (Madison, WI, USA). 2′7’-dichlorodihydrofluorescein diacetate (H_2_DCFDA) was purchased from Molecular Probes (Eugene, OR, USA). RIPA lysis buffer was purchased from Biomax (Gyeonggi, South Korea). Dako Fluorescence mounting medium was purchased from Agilent (Santa Clara, CA, USA). Chloroquine diphosphate (Cat. #C6628) and luteolin (Cat #L9283) were purchased from Sigma (USA). Ammonium chloride 99 + % pure (Cat #123,340,250) was purchased from Acros Organics (Geel, Belgium). Quercetin dihydrate was purchased from MP Biomedicals (Santa Ana, CA, USA). Rapamycin (Cat #HY-10219) was purchased from MedChemExpress (NJ, USA).

### Cell culture

The mouse hippocampal neuronal HT-22 cells were a generous gift from Prof. David Schubert (The Salk Institute, San Diego, CA, USA). SH-SY5Y neuroblastoma cells were purchased from a cell line service (Heidelberg, Germany; Catalogue number 300154). Neuro-2A cells were obtained from the Health Science Research Resources Bank (Osaka, Japan). HT-22 and Neuro-2A cells were cultured in Dulbecco's Modified Eagle's Medium (DMEM) supplemented with 10% fetal bovine serum, 100 U/ml penicillin, and 100 µg/ml streptomycin. SH-SY5Y cells were cultured in F12/DMEM (F12/DMEM 1:1 mixture) supplemented with 10% fetal bovine serum, 100 U/ml penicillin, and 100 µg/ml streptomycin. The cells were incubated at 37 °C in a humidified atmosphere with 5% CO_2_. The culture medium was changed every 3 days, and the cells were grown until they reached 80–85% confluence for the experiments. The cells passage between 10 and 25 were used.

### Cell viability assay

To evaluate the cell viability, we employed the 3-(4,5-dimethylthiazol-2-yl)-5-(3-carboxymethoxyphenyl)-2-(4-sulfophenyl)-2H-tetrazolium (MTS) assay. HT-22, Neuro-2A, and SH-SY5Y cells were seeded in 96-well plates at a density of 3,000 cells per well and allowed to adhere for 18–24 h. Subsequently, the cells were pre-treated with *A. lebbeck* leaf extract (2.5 µg/ml-100 µg/ml), quercetin (10 µM), and luteolin (5 µM-50 µM) for 24 h, followed by exposure to glutamate (5 mM) in complete medium. Incubation was carried out at 37 °C in a 5% CO_2_ incubator with a humidified atmosphere for 18 h. Next, 20 µl of MTS CellTiter 96® Aqueous one solution reagent (Promega, Madison, WI, USA) was added, and the cells were further incubated for 1 h. The absorbance was measured by a microplate reader (Multiskan™ FC Microplate Photometer, Thermo Scientific, Waltham, MA, USA) at 490 nm. The percentage of cell viability was calculated by comparing the results to the control group. Additionally, for qualitative assessment of cell morphology, the Nikon Eclipse Ti-U inverted microscope was employed.

### Lactate dehydrogenase assay

To assess cytotoxicity, the release of lactate dehydrogenase (LDH) enzyme from damaged cells was measured. HT-22 cells were seeded overnight in 96-well plates at a density of 3,000 cells per well. The cells were then pre-treated with quercetin (10 µM) and luteolin (5 µM-50 µM) for 24 h, after which they were exposed to glutamate (5 mM) in a complete medium. The incubation was carried out at 37 °C in a 5% CO_2_ incubator with a humidified atmosphere for 18 h. After the incubation period, 50 µl of supernatant was transferred to a new 96-well plate. The CytoTox 96® non-radioactive cytotoxicity assay (Promega, Madison, WI, USA) was employed for analyzing cell cytotoxicity. Briefly, a lysis buffer was added and incubated for 45 min to serve as a cell lysis control. For LDH enzyme measurement, 50 µl of cytotoxic reagent was added to the aforementioned 96-well plate, and the mixture was incubated for 30 min at room temperature. The reaction was halted by adding 50 µl of stop solution. Subsequently, absorbance was measured at 490 nm using a microplate reader (Thermo Fisher Scientific, Waltham, MA, USA). The percentages of LDH release were calculated and compared to the control group.

### Intracellular ROS

H_2_DCF-DA was utilized to measure intracellular ROS levels^66^. HT-22 cells were seeded in 48-well plates at a density of 8,000 cells per well. Following a 24 h pre-treatment with quercetin (10 µM) and luteolin (5 µM-25 µM), the cells were exposed to glutamate (5 mM) in a complete medium. The treated cells were incubated at 37 °C in a 5% CO_2_ incubator with a humidified atmosphere for 18 h. After the incubation period, 10 µM H_2_DCF-DA and 1 µM Hoechst were added to the cells and incubated at 37 °C for 30 min. The stained cells were washed twice with cold Hank’s balanced salt solution (HBSS). The fluorescence of dihydroethidium (DHE) was analyzed using a CX7 LZR high content screening (HCS) platform (Thermo Fisher Scientific, Waltham, MA, USA).

### Mitochondria ROS level (Mitosox)

HT-22 cells were seeded in a 48-well plate at a density of 8,000 cells per well. The cells were treated with quercetin (10 µM) and luteolin (5 µM-25 µM) for 24 h, and subsequently exposed to 5 mM glutamate in a complete medium for 18 h. The cells were stained with a specific mitochondria superoxide indicator (MitoSOX-red) (Molecular Probes, Eugene, OR, USA) at a concentration of 5 µM for 10 min. Subsequently, the stained cells were washed twice with cold HBSS. The fluorescence of mitochondria superoxide was analyzed using a CX7 LZR high-content screening (HCS) platform.

### Apoptosis assay

PE-Annexin V/7-AAD staining was performed on HT-22 cells that were seeded in 6-well plates at a density of 100,000 cells per well and allowed to adhere for 18–24 h. The cells were pre-treated with luteolin (5 µM-25 µM) for 24 h. Following this pre-treatment, the cells were exposed to glutamate for 18 h. Subsequently, the cells were harvested from the plate, washed with PBS, and resuspended in 100 μL of 1X Annexin V binding buffer. They were then incubated in the dark at room temperature with 5 µL of PE Annexin V and 5 µL of 7-AAD viability staining solution for 15 min. Then, the cells were diluted with 400 μL of binding buffer. For each experiment, unstained and single-channel controls were used to calculate compensation. Flow cytometry analysis was performed using a Cell sorter SH800S (Sony Biotechnology, San Jose, CA, USA).

### Western blotting

Cells were washed with cold PBS and then lysed on ice using pre-cooled RIPA lysis buffer containing protease inhibitors (Biomax, Gyeonggi, South Korea). The proteins (25 µg) were loaded into each lane of an 8–12% acrylamide gel (Biosesang, Gyeonggi, South Korea). After the separation step, the proteins were transferred to PVDF membranes (GVS, Bologna, Italy) and blocked with 5% nonfat dry milk in Tris-Buffered saline with 0.1% Tween 20 detergent (1X TBS-T) for 1 h. Primary antibodies LC3B 1:5000, BNIP3L/NIX (D4R4B 1:1000), Beclin-1 (D40C5 1:1000), Phospho-mTOR (ser2448) (D9C2 1:1000), mTOR (7C10 1:1000), Raptor (24C12 1:1000), Phospho-S6 Ribosomal Protein (Ser235/236 1:1000), Phospho-4E-BP1 (Thr37/46) (236B4 1:1000) were used to probe the target proteins at 4 °C overnight. Next, the proteins were probed with an HRP-conjugated secondary antibody (1:5000, Invitrogen, USA) at room temperature for 1 h. Finally, the proteins were detected using a SuperSignal™ West Pico PLUS Chemiluminescent Substrate (Thermo Fisher Scientific, Waltham, MA, USA), and the protein analysis was performed using NIH ImageJ.

### mtDNA analysis

The mtDNA copy number was analyzed according to previously reported^67^. To assess the mtDNA copy number, total genomic DNA was extracted by AccuPrep® Genomic DNA extraction kit (Bioneer, Daejeon, South Korea) according to the manufacturer’s instructions with the mouse mitochondrial genome. Since 16S rRNA represents a stable fraction (less prone to deletions)^68^, the 16S rRNA was selected to examine the relative copy number of mitochondrial DNA (mtDNA) and nuclear DNA (nDNA). Hexokinase 2 (HK2), a gene encoded in the nucleus, was specifically selected as a nuclear DNA (nDNA) for normalization (Table 1). The mtDNA expression was calculated by comparing the expression of 16S rRNA DNA to HK2 DNA expression. The mtDNA/nDNA ratio was calculated using the ΔΔCt method by calculating the number of mtDNA per nDNA. The Ct values, obtained from the qPCR machine software, represent the mean of triplicate Ct values for each DNA sample.

**Table 1:** Primer for mtDNA copynumber analysis.

Genes	Sequence of primer
*16S rRNA* forward	5′- CCGCAAGGGAAAGATGAAAGAC-3′
*16S rRNA* reverse	5′- TCGTTTGGTTTCGGGGTTTC-3′
*HkII* forward	5′- GCCAGCCTCTCCTGATTTTAGTGT-3′
*HkII* reverse	5′- GGGAACACAAAAGACCTCTTCTGG-3′

### Mitophagy and mitochondrial morphology analysis

HT-22 cells were placed on coverslips in a 6-well plate. The cells underwent the treatment as outlined previously. Each well was stained with 50 nM lysotracker deep red and 150 nM mitotracker orange CMTMRos. After rinsing with PBS, the cells were fixed with 4% paraformaldehyde at room temperature for 15 min. Subsequently, cells were stained with 1 µM Hoechst for 10 min. After rinsing with PBS, stained cells were mounted with Dako fluorescence mounting medium. Finally, the slides were examined using a confocal laser scanning microscope (LSM 800) from Carl Zeiss, Germany. The colocalization was analyzed by Image J software, the JACoP plugin. In addition, mitochondrial morphology and branching were analyzed by Image J software, the MiNA plugin.

### Mitochondria membrane potential

The cells were seeded in a 48-well plate at a density of 8,000 cells per well and cultured under the same conditions as mentioned previously. After glutamate treatment for 12 h, the cells were subjected to staining with MitoTracker® Orange CMTMRos (25 nM) and incubated at 37 °C for 30 min. Subsequently, the cells were washed three times with PBS and fixed using 4% paraformaldehyde (PFA) for 10 min at room temperature (RT). For nucleus staining, the cells were stained with DAPI (10 μg/ml) for 10 min at RT and washed with PBS. The fluorescence intensity was quantified and visualized using the Thermo Scientific™ CellInsight CX7 high-content screening platform.

### PCR array assay

Total RNA was extracted from the cells using Trizol reagent (Thermo Scientific, Waltham, MA, USA), and the RNA concentration was measured using a Nabi- UV/Vis Nano Spectrophotometer from Microdigital, Gyeonggi, South Korea. The RNA was then converted into complementary DNA (cDNA) through reverse transcription using the Verso cDNA synthesis kit from Thermo Scientific, USA. The real-time RT2 profiler mouse autophagy PCR array (QIAGEN, Cat. no. PAMM-084Z) was used to analyze the autophagy-related gene expression. The 96-array plate consists of controls for genomic DNA contamination, reverse transcription and positive PCR controls. Glyceraldehyde-3-phosphate-dehydrogenase (GAPDH) served as the reference gene for the assay. CT values were recorded in an Excel file to generate a table of CT values, which was then uploaded to the data analysis web portal at http://www.qiagen.com/geneglobe. The samples included both control and test groups, and the CT values were normalized using a manual selection from the full panel of reference genes.

### Data analysis

Each experiment was performed at least three independent experiments, and the values are expressed as the mean ± standard error of the mean (SEM). One-way analysis of variance (ANOVA) was used for the evaluation of the statistical significance with post hoc Dunnett’s test and Bonferroni. A *p*-value of less than 0.05 was considered statistically significant.

### Supplementary Information

Supplementary Figures.

## Data Availability

The datasets used and/or analysed during the current study are available from the corresponding author on reasonable request.

## Abbreviations

ALE: Albizia lebbeck leaf extracts
ROS: Reactive oxygen species
H_2_DCF-DA: 2,7-Dichlorodihydrofluorescein diacetate
BNIP3L/NIX: BCL2/adenovirus E1B interacting protein 3-like
mtDNA: Mitochondrial DNA
nDNA: Nuclear DNA
mTORC1: Mammalian target of rapamycin complex 1
CQ: Chloroquine
UVRAG: UV radiation resistance associated
